# Supplementary material for: An RNAi screen of Rab GTPase genes in C. elegans reveals that somatic cells of the reproductive system depend on rab-1 for morphogenesis but not stem cell niche maintenance
Source: bioRxiv. 2024 Dec 6:2024.12.03.626641. Preprint. [Version 1] doi: 10.1101/2024.12.03.626641 (PMC11642880; doi:10.1101/2024.12.03.626641)

bioRxiv preprint doi: https://doi.org/10.1101/2024.12.03.626641; this version posted December 6, 2024. The copyright holder for this preprint (which was not certified by peer review) is the author/funder, who has granted bioRxiv a license to display the preprint in perpetuity. It is made available under aCC-BY 4.0 International license.

Supplemental Figures

Figure S1. Locations of *lag2p::mNG::F2A::rde-1* rescue transgene expression during development. Representative images of the strain bearing the *lag2p::mNG::PLC$^{\delta PH}$::F2A::rde-1* allele (with mutations in *rrf-3(pk1426)* and *rde-1(ne219)*) in the L1 (A), L2 (B-C), turning/late L3 (D-E), L4 (F), and Adult (G) life stages in which RNAi is presumably active in mNG+ cells (with the exception of neurons, which are not sensitive to RNAi (Calixto et al., 2010), seen along the ventral body wall in some images and in superficial circumferential projections in B). Single slices or projections through 0.5-2.5 microns from confocal z-stacks, DIC images merged with green fluorescence (right), and green fluorescence alone (grayscale, center and insets). Imaged with 0.5 micron step size, except (A), which was imaged with 0.2 micron step size. Greyscale images shown with fluorescence logarithmically scaled to enhance visibility of dim signal. Boxes show region of inset. Asterisks mark distal tip. (A) Somatic gonad progenitor cells Z1 and Z4 express mNG in L1 arrested larvae, with substantially more expression in Z4 than Z1; primordial germ cells Z2 and Z3 do not express the rescue transgene. (B) In the L2, cells of the sheath-spermathecal (SS) lineage (outlined in yellow dashed lines) are mNG+ early, possibly as residual expression from the somatic gonad precursor cells; these cells are no longer visible by the turning stage in L3 (brackets in D). (B') Projection through deeper Z-slices of the same sample in B. The anchor cell (AC) is mNG+ from the late L2 when it is first born. (C) SS cells are still faintly mNG+ at the time of AC invasion (inset). (D) By the time of DTC turning in the L3, no SS expression is observed, and signal at the future site of vulva formation (inset) is very dim. (E) As the turn is completed, the vulval precursor cells (inset) are mNG+. (F) The vulva remains mNG+ in L4 larvae and (G) adults. Scale bars = 20 µm.

Table S1. RNAi clones made for this study

C. elegans rab gene	Forward Primer	Reverse Primer	RNAi insert length*
glo-1	5'-CGTATTTGCCAAAAATTTGAACGCC-3'	5'-AGGCCAAGACCGATATGGGG-3'	364 bp
Y71H2AM.12	5'-GAACTCACTCACTAACAAGATCGTGC-3'	5'-GTCAATTCGCCTTCAATTATGGG-3'	276 bp
rab-14	5'-CGTATTTGGATTGGTCAAGC-3'	5'-AAATGACGGCTGCTCCTTAC-3'	499 bp

bioRxiv preprint doi: https://doi.org/10.1101/2024.12.03.626641; this version posted December 6, 2024. The copyright holder for this preprint (which was not certified by peer review) is the author/funder, who has granted bioRxiv a license to display the preprint in perpetuity. It is made available under aCC-BY 4.0 International license.

Figure S1. Locations of *lag-2p::mNG::F2A::rde-1* rescue transgene expression during development

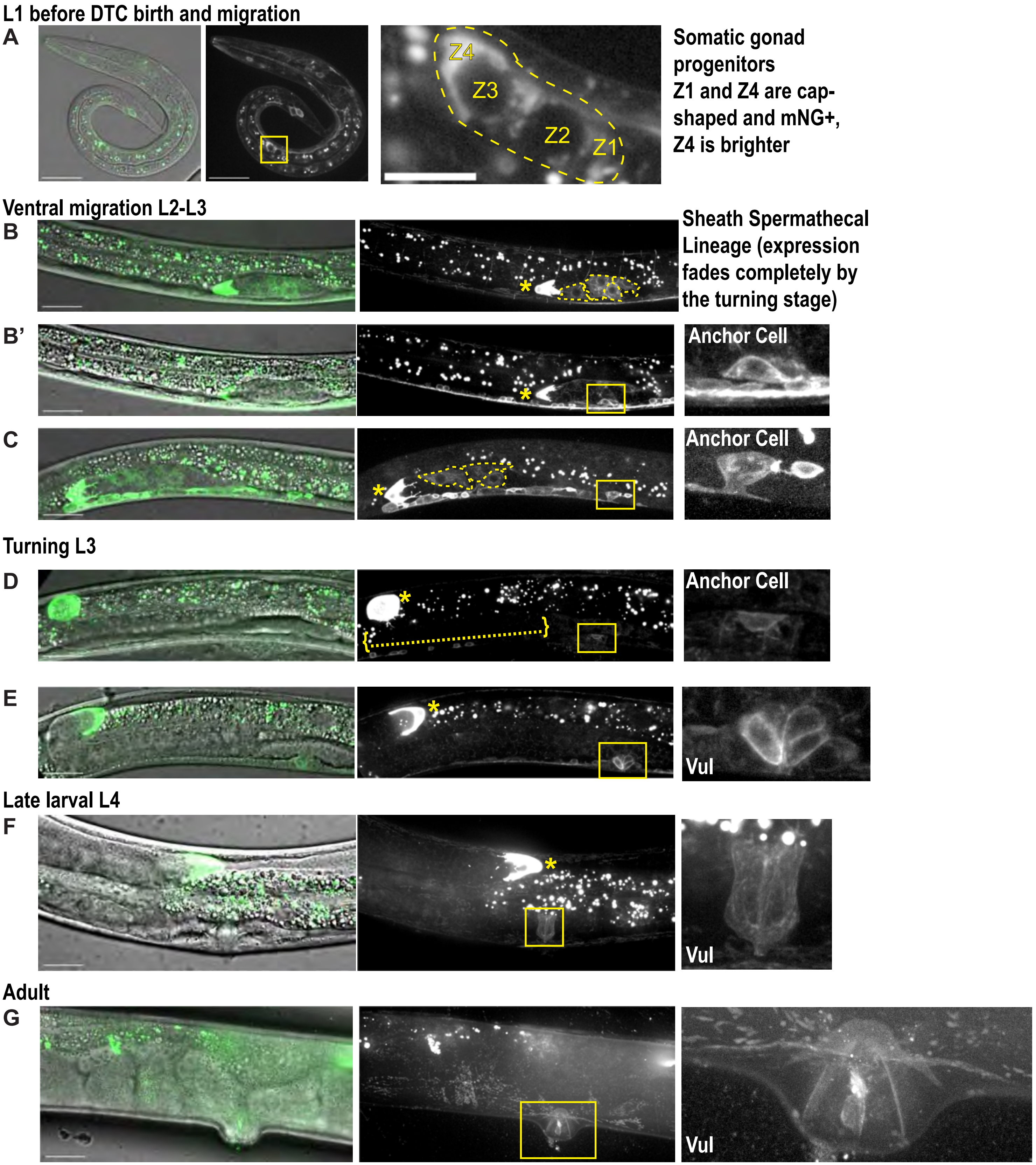

Supplement: Supplement 1 [file NIHPP2024.12.03.626641v1-supplement-1.pdf]
